# Design of a Wearable Vibrotactile Stimulation Device for Individuals With Upper-Limb Hemiparesis and Spasticity

**DOI:** 10.1109/TNSRE.2022.3174808

**Published:** 2022-05-17

**Authors:** Caitlyn E. Seim, Brandon Ritter, Thad E. Starner, Kara Flavin, Maarten G. Lansberg, Allison M. Okamura

**Affiliations:** Department of Mechanical Engineering, Stanford University, Stanford, CA 94305 USA; Department of Mechanical Engineering, Stanford University, Stanford, CA 94305 USA; Department of Computer Science, Georgia Institute of Technology, Atlanta, GA 30332 USA.; Department of Neurology, Stanford University, Stanford, CA 94305 USA.; Department of Neurology, Stanford University, Stanford, CA 94305 USA.; Department of Mechanical Engineering, Stanford University, Stanford, CA 94305 USA

**Keywords:** Accessibility, design, haptics, patient rehabilitation, stroke, upper limb, user centered design, wearable devices

## Abstract

Vibratory stimulation may improve post-stroke symptoms such as spasticity; however, current studies are limited by the large, clinic-based apparatus used to apply this stimulation. A wearable device could provide vibratory stimulation in a mobile form, enabling further study of this technique. An initial device, the vibrotactile stimulation (VTS) Glove, was deployed in an eight-week clinical study in which sixteen individuals with stroke used the device for several hours daily. Participants reported wearing the glove during activities such as church, social events, and dining out. However, 69% of participants struggled to extend or insert their fingers to don the device. In a follow-up study, eight individuals with stroke evaluated new VTS device prototypes in a three-round iterative design study with the aims of creating the next generation of VTS devices and understanding features that influence interaction with a wearable device by individuals with impaired upper-limb function. Interviews and interaction tasks were used to define actionable design revisions between each round of evaluation. Our analysis identified six new themes from participants regarding device designs: hand supination is challenging, separate finger attachments inhibit fit and use, fingers may be flexed or open, fabric coverage impacts comfort, a reduced concern for social comfort, and the affected hand is infrequently used. Straps that wrap around the arm and fixtures on the anterior arm were other challenging features. We discuss potential accommodations for these challenges, as well as social comfort. New VTS device designs are presented and were donned in an average time of 48 seconds.

## I. Introduction

THE American Heart Association projects that by 2030 approximately 3.9% of the US population will have had a stroke [1]. Stroke often leads to physical disability, and upper-limb disability is a key factor in self-sufficiency. Following stroke, 35-55% of people have diminished tactile perception in their hand or arm [2], and 50% have upper-limb motor disability [3] (40-50% of whom have spastic hypertonia [4], [5]). Technology-enabled techniques are needed to increase accessibility of therapy to this expanding population and to explore new methods for relieving post-stroke symptoms.

Spastic hypertonia can impair or prevent actions such as releasing a grasp and performing activities of daily living [6]. Spastic hypertonia can also lead to other impacts such as contractures and patient discomfort [7]. Impaired tactile perception can affect dexterity [8] or lead patients to be unaware of an injury [9]. Treatment of these symptoms is of significant clinical importance.

### A. Vibrotactile Stimulation

Vibrotactile or vibratory stimulation (VTS) may provide some symptom relief after stroke. The leading application of VTS focuses on reduction of spastic hypertonia. Vibrotactile stimulation is used clinically to relieve muscle tone [10], and prior work reports both acute and potentially durable reductions in spastic hypertonia while using vibrotactile stimulation [11]-[15]. For example, Marconi *et al.* found an average one-point reduction on the Modified Ashworth scale (MAS) after vibration of the spastic limb for 30 min. on 3 consecutive days in stroke [14]. Murillo *et al.* found average reductions of 1.1 points in MAS as well reductions in clonus, Hmax/Mmax ratio, and T wave amplitude during stimulation of the spastic limb in spinal cord injury [15]. Certain frequencies of vibration produce intense Ia spindle discharge. Ia discharge, which also occurs during muscle stretch, directly modulates muscle activity via the stretch reflex [16]. Ia input induces reciprocal inhibition of the antagonist to stimulated muscle, which is typically implicated as the mechanism underlying VTS for spastic hypertonia relief [12], [13]. Intensive Ia discharge also leads to increased presynaptic inhibition and Ia postactivation depression in the muscle, thought to diminish the sensitivity of the hyperexcitable stretch reflex in spastic hypertonia [15].

Other research has shown an association between application of vibrotactile stimulation and improved tactile perception [17]-[19]. For example, Estes *et al.* found improvements in Semmes-Weinstein monofilament exam (SWME) at all tested locations on the hand after vibrotactile stimulation of the hand for 2 hours, 5 days per week, for 8 weeks in incomplete spinal cord injury [18]. Seim *et al.* found longitudinal improvement in tactile perception assessed using the Semmes-Weinstein monofilament exam after 3 hours of daily finger stimulation over eight weeks in stroke [17]. Somatosensory input is known to drive cortical organization during development, learning, and rehabilitation [20], and vibrotactile input provides strong somatosensory input via cutaneous mechanoreceptors [21]. In the case of sensorimotor lesions, cutaneous input may induce central reorganization through intact peripheral nerves and residual sensorimotor connections – a possible mechanism for stimulation-linked improvements in impaired tactile perception. Others have also found improved Wolf Motor Function Functional Ability score and range of motion after or during stimulation of the limb [11], [14], [15]. Indeed, VTS is also applied as an adjunct to movement therapy [14], [18], [22], [23].

### B. The Importance of Wearability

Vibrotactile stimulation could provide a useful adjunct to therapy; however, current apparatus used to apply this stimulation have limitations: they are not mobile and they may require assistance to operate. For example, two of the most prevalent methods are whole body vibration (WBV), which requires participants to stand on a scale-like platform, and repeated focal muscle vibration (rMV), which uses stationary machines with pins to provide stimulation.

A wearable device would provide significant advantage to therapeutic vibrotactile stimulation. Since wearable devices can be closely coupled with the human body while the wearer performs unrelated tasks, a wearable device could provide stimulation during daily life rather than during designated therapy time. *Wearablility* enables more chances to use the device and thus more opportunities for adherence. Wearability supports freedom of the user when they want to leave the home and still get stimulation, or when they want stimulation while performing other tasks. Wearable VTS could uniquely be used in an assistive context, for example, to keep muscles relaxed during activity. A wearable design also allows longer durations and repetitions of vibrotactile stimulation, which have not yet been studied in clinical trials. Current laboratory studies typically apply this stimulation for periods of 5-30 minutes [11]-[15], [22], [24]-[28]. Intensity (therapy time) is known to be associated with improved rehabilitation outcomes [29]-[31]. Some preliminary results display promise when researchers applied a longitudinal stimulation schedule, requiring visits 3-5 days per week for 1-4 weeks (spastic hypertonia) [11], [14] and 3-7 days per week for 1-4 weeks (sensorimotor retraining) [25], [26]. Participants had to visit the laboratory to use stimulation equipment.

There are also requirements to achieve wearability. The efficacy of a medical device depends both on therapeutic settings and device use (adherence). Devices that are unused cannot result in therapeutic benefit. The Technology Acceptance Model discusses determinants of technology adoption [32], [33], the two cornerstones being ease of use and perceived usefulness. For wearable devices, these factors are influenced by breakage, dimensions and weight [34], perceived comfort [34], [35], and interaction time [33], [36], [37]. Individuals with limb disability require additional consideration. It is known that spastic hypertonia and hemiparesis can inhibit activities such as dressing, even when given assistance from a caretaker [38], [39]. Donning and doffing are the primary interactions users have with a passive stimulation device; thus these interactions are a driving factor in the perceived ease of use for the device. Accommodations that let affected individuals put on (don) and remove (doff) the device themselves enable participation in therapy for those without caretakers and independence for all involved.

Prior work calls attention to the need for user-centered and accessible design in rehabilitation technology [40]-[42] and wearable technology [43]-[45], but does not describe potential accommodations in detail. A better understanding of how to reduce time, difficulty, and confusion would allow system designers to make more usable devices. Well-designed devices allow clinical trials to focus on clinical efficacy, rather than adherence and logistical issues.

### C. The VTS Glove and Clinical Study

We designed an initial wearable vibrotactile stimulation device, the VTS Glove, and conducted an 8-week clinical study in chronic stroke [17]. The aims of this work were to both measure clinical outcomes, and gather data on device design and usability.

The device was designed to provide cutaneous input to the fingers to evaluate its longitudinal effect on diminished tactile perception after stroke. Participants were asked to wear the device for three hours per day. Clinical data revealed a significant change in tactile perception and spastic hypertonia associated with the longitudinal stimulation from the VTS Glove (details of the clinical work can be found in Seim *et al.* [17]).

**Fig. 1.**
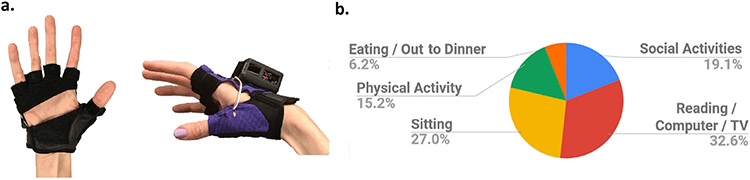
(a.) The VTS Glove, which provides wireless and wearable vibrotactile stimulation. (b.) Breakdown of activities performed while wearing the stimulation device, reported by sixteen persons with stroke in the clinical study.

Adherence, durability, and comfort of the VTS Glove indicated positive results (details of the design findings are provided in the Supplementary Material). Some participants described wearing the glove to activities such as movies, church, brunch, family gatherings, riding a bicycle, exercising, and picnics (activity breakdown in Figure 1b). However, data also revealed the need for revision of the device.

The device design (Figure 1a) was based on prior work in which vibrotactile “MusicTouch” gloves were used to teach piano to patients with impaired hand dexterity from incomplete spinal cord injury (iSCI) [46]. The MusicTouch glove had some features suitable for users without disability and individuals with incomplete spinal cord injury, namely: the glove was fingerless for fit, and palmless for sanitation and to allow wearing while operating a manual wheelchair [18]. The fingerless glove design worked well for this iSCI population – who had mild motor dysfunction (with no spasticity and hypertonia) from partial spinal cord injury. However, during the VTS Glove clinical study, participants with stroke struggled to get the glove on and off their affected hand. Between their fifth and sixth week of participation, three participants were timed during the donning process. The three randomly selected participants recorded an average time of 303 seconds (approx. 5 minutes) to don the glove after participating for at least four weeks. Eleven out of sixteen participants reported difficulty with the don and doff process. Participants with spastic hypertonia or weakness had difficulty placing each finger in one of the glove sleeves, even with help from a caregiver. They *could not extend or insert their fingers*. Instead, their able hand was used to manipulate the affected fingers for extension and insertion. This prevented use of the able hand to simultaneously don and secure the device.

Such difficulty could result in strain and frustration, and may dissuade prospective users from adopting a new technology [33]. Prior work has also identified the don and doff process as a critical challenge in wearable rehabilitation devices. Louie *et al.* [32] identified a gap in wearable sensing technology for individuals with stroke. Their work revealed donning/doffing with one arm as one of five design features that participants considered necessary, along with size, weight, appearance, and cleaning. They also found design flaws, such as taking too long to set up, as one of three key potential barriers to adoption. Setup time and learning curve was identified by Elnady *et al.* as one of three potential challenges for users of upper-limb wearable robotic devices [47], and Moineau *et al.* found easiness to don and doff as one of four challenges to wearable FES garments after stroke and SCI [48].

### D The Next Phase of VTS Devices

The results of the clinical study using the VTS Glove were particularly promising for the reduction of spastic hypertonia [17] and additional study is needed. Revision of the VTS Glove is essential to enable the next stage of experiments.

VTS Phalanx, is a revised form of the VTS Glove designed in this manuscript to apply dorsal phalanx stimulation as in prior work. In addition, here we designed wearable devices for two additional stimulation zones on the upper limb. The VTS Armband was designed to wrap around the musculotendinous regions of the forearm. Finally, the VTS Palm was designed to stimulate the volar hand. None can fully isolate a single type of tissue while maintaining a wearable design, but each of these designs form the basis for further research on stimulation at different locations: Specifically, the optimal stimulation approach for spastic hypertonia is still unknown. Stimulation at the spastic muscle belly is of significant interest in prior work [11]-[15], [24], and no work has compared VTS at the hand vs. VTS at the muscle belly, or the longitudinal application of wearable VTS at the muscle belly. Future experiments could also use a wearable VTS device to evaluate the impact of antagonist vs. agonist muscle stimulation in this context. Furthermore, the use of VTS to improve impaired tactile perception is also of clinical interest. It is unknown if stimulation is more effective than dorsal hand stimulation when applied directly to sensing areas of the hand, such as the glabrous skin of the volar hand (which is extremely dense in cutaneous sensory receptors [49]). Stimulation at the palm could reach a large area of glabrous skin while leaving the fingers free.

Iterative evaluation and revision of these devices can improve each design, and can also inform the design of wearable and arm-mounted devices for this population more generally. **The aims of this work are:**

- Design wearable VTS devices for people with stroke based on initial findings from a 8-week study using a vibrotactile glove in stroke – to enable further research on clinical impact.
- Understand features that influence interaction with a wearable device by individuals with diminished upper-limb function.

## II. Methods

The clinical study of the VTS Glove (Section I.C. and Supplementary Material) revealed the need for design accommodations for wearable VTS devices. We performed a follow-up study to create improved devices, and to identify the factors that influence persons with stroke experience using an upper-limb wearable device.

Lakshminarayanan *et al.* surveyed individuals with stroke about a wearable rehabilitative device. When participants were asked about acceptable don/doff duration, 3/10 chose < 5 min. and 6/10 chose < 1 min. [50]. Participants preferred shorter donning times. We aim for the donning time of a VTS device to approach 1 minute. As an initial step we hypothesized that, with revision, the new device prototypes could be donned in less than five minutes (300 s).

### A. Study Design

An iterative design-and-test methodology was used to evaluate three new prototypes. This structure enabled multiple rounds of revision and introspection that could not be achieved in a single-round study. (This is in contrast to an evaluation study using ranking measures such as the NASA TLX [51]).

**Fig. 2.**
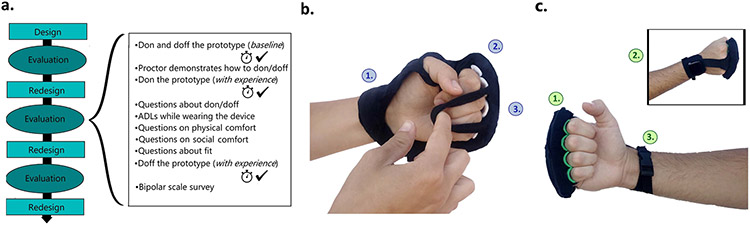
(a.) Study structure. (b.) VTS Phalanx version V1. 1. A design resembling fingerless gloves with the palm exposed. **2.** Rigid arches create a place for each finger without inserting each finger individually. **3.** The two straps are capable of wrapping over flexed fingers without requiring finger extension. (c.) VTS Phalanx version V4. **1.** The single strap over the fingers can be donned with the anterior arm facing away (no supination). **2.** A T-strap design reduces fabric coverage while providing a path for wires to run between circuitry at the wrist and actuators at the fingers. **3.** A buckle closure enables strap adjustment using one hand.

There were three rounds of design and evaluation in this study (as shown in Figure 2a). There was one final prototype design made (V4) after evaluation round 3, so that a prototype could be made in view of the feedback from round 3. Two or three persons participated in each round. Participants first signed a consent form and removed any jewelry and watches on the affected arm. Next, the voluntary active range of motion (AROM), Modified Ashworth scale ratings, and other demographics were recorded by a trained proctor. These data indicate the participant’s level of dexterity and can be found in the Participants section and in table form in the Supplementary Material. Each study visit was one hour in length. During their visit, each participant was asked to evaluate the three prototypes one at a time in a randomized order. All procedures (see Section II-E) were repeated for each prototype. Each visit was recorded using a video camera and later transcribed verbatim. Study investigators had no relationship to the participants. Participants were assigned an alphanumeric identifier to anonymize data.

### B. Participants

Participants were eight individuals with chronic stroke (ages 48-89, 6 months to 4 years post stroke, 5 male/3 female). Individuals with chronic stroke and impaired hand function were recruited through a local clinic according to the following inclusion criteria: history of stroke, no individual finger control on their affected hand, and limited range of motion in the fingers (< 90° of voluntary extension at metacarpophalangeal joints (MCP)). 5/8 participants had spastic hypertonia. Active range of motion for this group was: MCP flexion 5°-55° (Mean=25°), MCP extension from flexed 0°–40° (Mean=12°), wrist flexion 0°–40° (Mean=19°), wrist extension from flexed 0°–35° (Mean=10°), elbow flexion 10°–85° (Mean=57°), elbow extension from flexed 0°–80° (Mean=43°). Individuals with aphasia were excluded because they could not provide verbal feedback. The setting was a university research lab, and the study was conducted in August 2019. All participants provided written consent, and the protocol was approved by the Stanford University Institutional Review Board.

### C. Design Goals

Design goals were identified for the new prototypes. Goals G1 and G2 were based on the results of the clinical study. G3-G6 are general features of wearable device design relevant to both users without disability and users with motor impairments.

#### 1) G1: Fast to Don/Doff With Hand Spasticity or Weakness:

Participants in the clinical study struggled to don the glove onto their affected hand. Reducing the interaction time is known to improve user experience and increase adoption of new technologies [32], [52]. We aim to reduce the time it takes to put on and remove the device, even when individuals have clenched hands due to spasticity and hypertonia, or limp fingers due to hemiparesis.

#### 2) G2: Reliance on One Hand:

Stroke affects primarily one side of the body [53]. When function is impaired on the affected side, the able hand is responsible for interacting with and adjusting equipment such as a wearable device.

#### 3) G3: Easy to Use and Understand:

Device design can encourage correct use. Correct use includes: how tight the device is worn and if the parts of the body are inserted correctly. Reducing confusion can also improve perceived ease of use, which is associated with technology acceptance [33].

#### 4) G4: Physically and Socially Comfortable:

Comfort of a wearable computing device includes physical and social components, because the device may be worn throughout daily life [54]. If users find a device uncomfortable, they are less likely to wear it [34].

#### 5) G5: Fit:

A one-size-fits-all, adjustable device allows more rapid production than custom devices or multiple sizes. A good fit also enables sensors and actuators to make reliable contact with the skin.

#### 6) G6: Durability:

For our application of wearable vibrotactile stimulation, devices may be worn for extended periods of time during the user’s daily activities. Repeated donning/doffing will result in strain on the user and wear on the device, so designs must be robust. If devices are used in rural or underserved areas, they must remain functional or be easily repaired.

### D. Initial VTS Device Designs

Based on the design requirements, initial designs were created for use in this study. Each design aims to apply four or more coin-shaped actuators to the body at the designated location (though no stimulation was applied during this study). All were designed to dissuade insertion or extension of the fingers, in view of the first study’s results. Each device design was fabricated into a non-stimulating prototype to allow participants to interact with design features. Prototypes also included an acrylic box at the location where electronics can be mounted.

#### 1) VTS Phalanx:

Like the VTS Glove, this device (Figure 2b) is intended to provide vibrotactile simulation to the dorsal proximal phalanx. The dorsal phalanx location presented here is based on prior work [17], [18], [46]. Fabric attaches with Velcro around the distal forearm and extends to the dorsal phalanges, leaving the palm exposed. This design maintains a similar shape to the VTS Glove; however, the glove’s finger sleeves are replaced with two straps that wrap over the fingers. These straps can be stretched over flexed fingers, so the design can be donned without finger extension. A rigid arch rests on each dorsal phalanx to keep the fingers in place without inserting the fingers individually. These arches are mounted on a foam bar that flexes to accommodate a variety of hand sizes.

#### 2) VTS Armband:

**Fig. 3.**
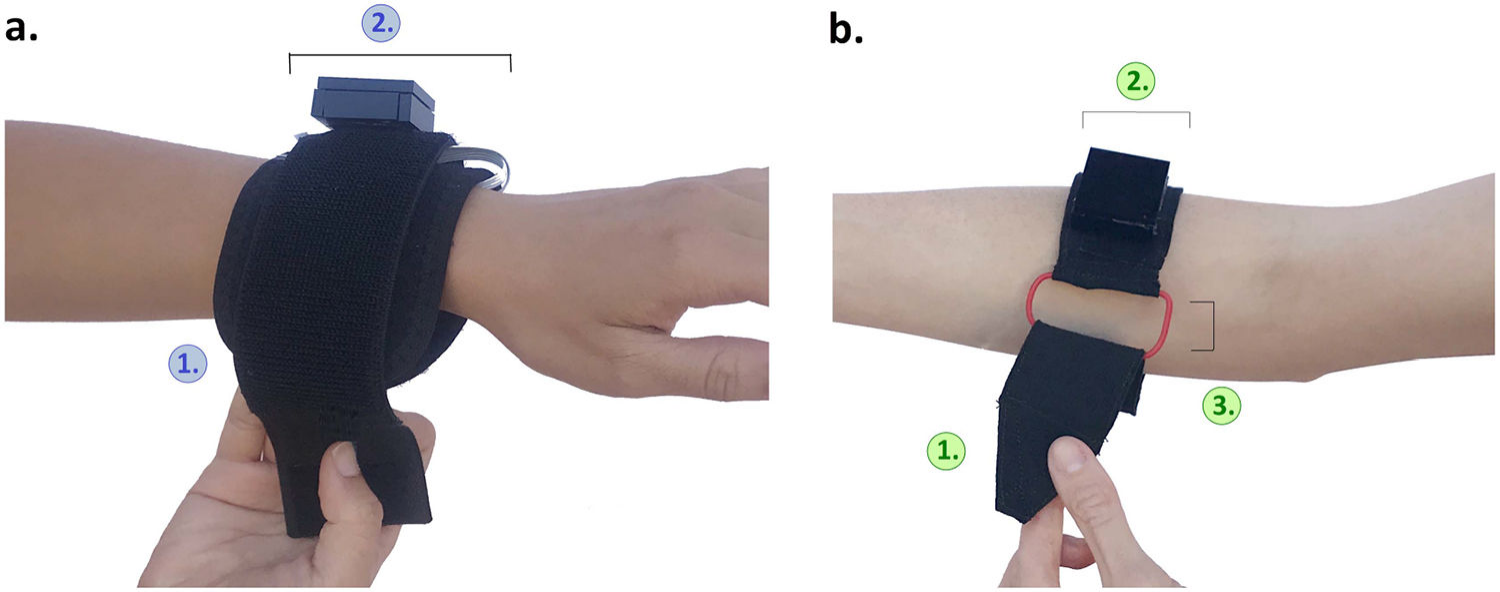
VTS Armband version V1 and version V4. (a.) **1.** The rectangular armband wraps around the arm and attaches via Velcro. **2.** The band has a width of 3 inches and circuitry can be mounted on or in the armband. (b.) **1.** The rigid and tapered end of the armband is designed to slip through the 0.75 inch buckle frame. **2.** The armband is 2 inches wide. **3.** A cinch buckle enables users to secure the device using one hand.

The Armband (Figure 3a) wraps around the forearm and allows stimulation to be applied to the muscle belly of the extrinsic hand muscles. The 3 inch-wide band is 15 inches long for fit around different size arms. The fabric band is attached using Velcro, and electronics can be mounted on or inside the band.

#### 3) VTS Palm:

**Fig. 4.**
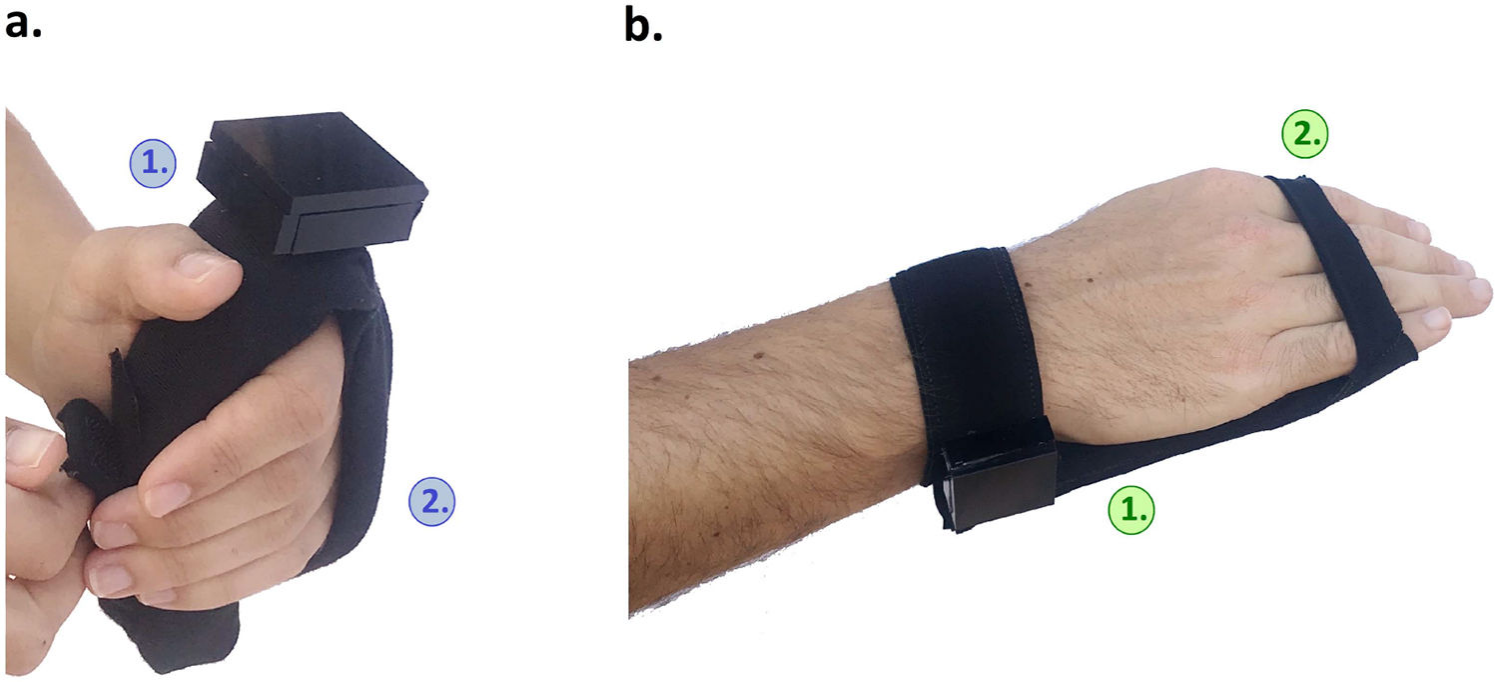
VTS Palm version V1 and version V4. (a.) **1.** The affected hand can be placed around the rod-shaped device. **2.** An adjustable strap slips over the fingers. (b.) **1.** The brace-like design contains foam which conforms to the palm when fingers are extended or flexed. **2.** A single strap secures the fingers.

This design (Figure 4a) enables stimulation at the volar hand (palm). This prototype has a graspable design. The body of the device is a foam rod (4 inches). The foam rod is reinforced inside with a semi-rigid tube that provides structural support.

### E. Procedures and Measures

Measures were chosen to provide data on each design goal. There were four types of measures used in this study: timed tasks, binary correctness measures, bipolar scale questions, and interview transcripts. The order of procedures is shown in Figure 2a; where timed tests are indicated by a clock and rating the correctness of a task is indicated by a check mark.

- The participants were asked to don and doff the prototype while sitting at a table. The proctor observed and asked follow-up questions (given in G1 below), made note of challenges, and rated the correctness of how the device was donned (see G3).
- The participant was next asked to demonstrate three functional tasks while wearing the prototype (writing on a clipboard, holding something in their lap, and walking). This provided another opportunity for observations and feedback (see G2).
- Next, questions about physical and social comfort (see G4) and questions about fit (see G5/6) were asked while the participant wore the device.
- A bipolar scale survey was given at the end of the measures and included questions about comfort and confusion (for all questions and responses, see Supplementary Material, Figure S5).

Interview guides were carefully designed according to qualitative study design principles [55], [56] to prompt insightful discussion without leading participants. Questions were standardized across prototypes and participants, and proctors could ask follow-up questions to gather more information. We used SRQR reporting guidelines [57].

#### 1) G1: Fast to Don/Doff With Hand Spasticity or Weakness:

Participants were first asked to don and doff the prototype. Their speed was measured using a stopwatch. They were not asked to rush. The proctor said “Could you please try putting on the device? Let me know when you are done by saying ‘I’m done.’ The proctor then asked the following questions:

“What do you think?”

“Was it easy or difficult to get on?”

“How would you make it easier to don?”

Next the proctor demonstrated how to don and doff using their own hand. The participant then donned the device again and provided feedback on the challenges and successful features. Participants later doffed the prototype at the end of the session. These tasks were timed.

#### 2) G2: Reliance on One Hand:

The don/doff tasks were used to demonstrate participants’ ability to interact with the device while primarily relying on their unaffected hand. After donning the prototype for the second time, participants were also asked to demonstrate three tasks of daily living to reveal challenges and elicit additional participant feedback. These tasks were: writing on a clipboard, holding something in their lap, and walking.

#### 3) G3: Easy to Use and Understand:

Each time that participants donned the device, their correctness was recorded as a binary measure. To be considered correct, the device must be in the correct position and adjusted securely. After removing the prototype at the end of the session, participants were given a bipolar scale survey to rate their level of confusion when using the prototype.

#### 4) G4: Physically and Socially Comfortable:

While wearing the device, participants were asked a series of interview questions about comfort that included both physical and social factors. Three examples are shown below.

“What activities would you do while wearing this?”

“What do you think about wearing this in public?”

“Do any parts of the device cause discomfort?”

The bipolar scale questionnaire, given at the end of the session, also included three questions regarding physical and social comfort.

#### 5) G5 and G6: Fit and Durability:

Before doffing the prototype for the second time, participants were asked “How secure does the device feel?” The study proctor also recorded observations of fit, breakage, or strain throughout the study and these observations are reported in the discussion section.

#### F Data Analysis

Thematic content analysis was performed on the participants’ transcribed verbal responses. Between each round, transcripts of the interviews were read and relevant content was then broken up into parts based on topic, independently by two of the investigators. These comments were separated into general feedback (i.e. “this is comfortable”) or actionable insights. Similar comments were tabulated together to form initial codes. This tabulation continued throughout all study rounds (see Supplementary Material, Figure S4). All comments were then discussed and checked against the original transcripts. Next, actionable codes were discussed to determine revisions for next round. Revisions were made to address codes supporting a design change with no contraindications.

After three rounds of feedback, each investigator grouped codes to define themes of perspectives across all participants and prototypes. These themes were then discussed among the two investigators with reflection on potential bias from individual participants and/or the investigators. Transcript data was used in conjunction with bipolar scale ratings and quantitative measures to add triangulation to the findings. Numerical values were assigned to bipolar scale responses and the Wilcoxon signed-rank test was used to compare responses within the group between questions.

## III. Results

**Fig. 5.**
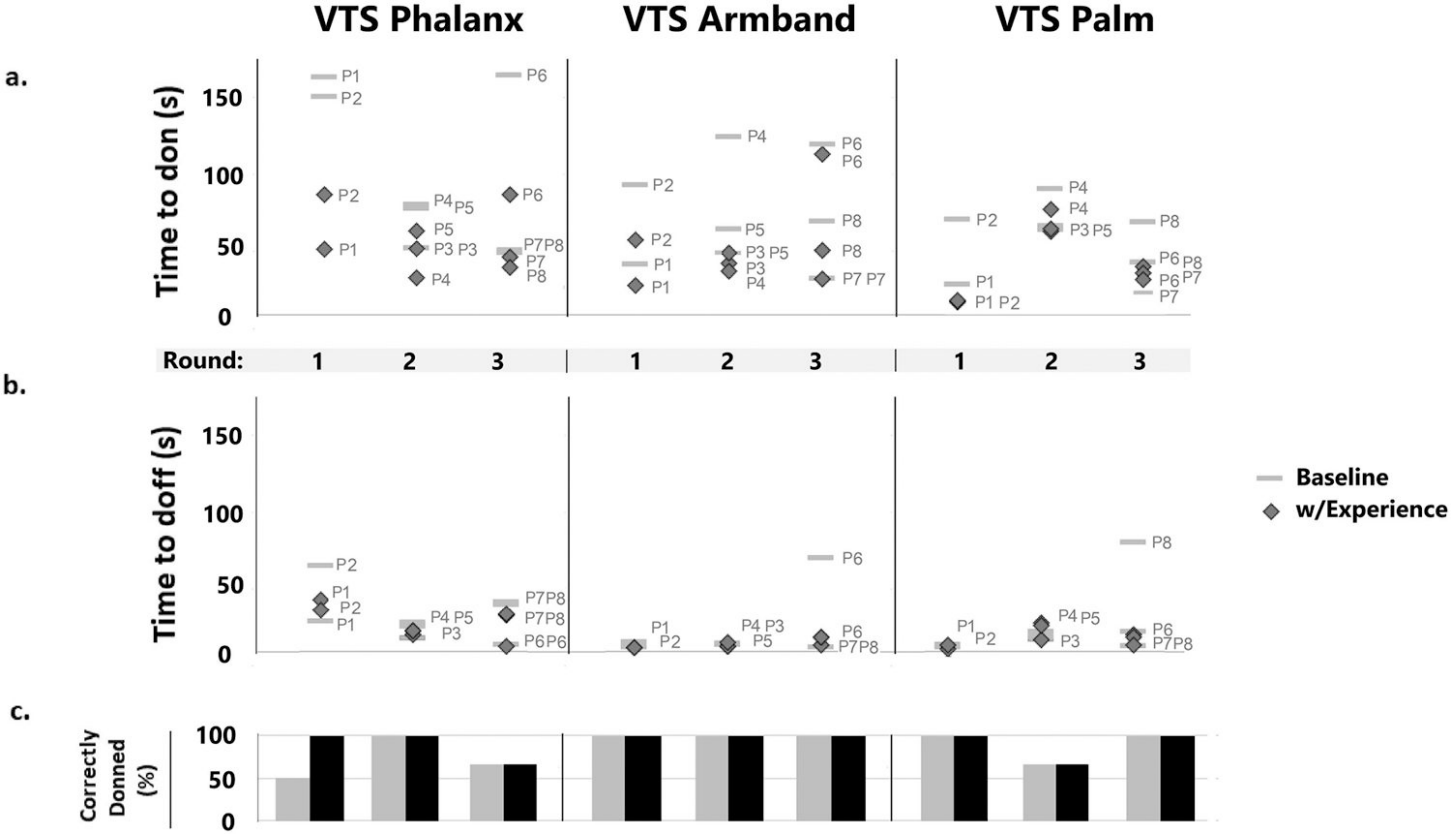
Quantitative data for each design and each round in the study. Participant numbers are listed by each data point. (a.) Time required to don the prototype for each participant. Each stack of markers represents one round in the study. (b.) Time to doff the prototype. (c.) Percent of participants who correctly donned the prototype for each round and each device.

Donning time for all prototypes was as follows: *VTS Phalanx* 31-172s, Mean (with experience) = 57 s; *VTS Armband* 15-120 s, Mean = 41 s; *VTS Palm* 11-90 s, Mean = 39 s. Data from the timed tasks and correctness measures are provided in Figure 5. A final version of each prototype (V4) was made in view of the feedback from round 3 and is displayed in the right of Figures 2 3, and 4. All transcripts were analyzed and data are presented below.

### VTS Phalanx:

The final VTS Phalanx (Figure 2c) uses a t-strap design to connect the fingers and distal forearm, so that more of the hand is uncovered. Electronics can be mounted securely at the wrist, and actuators can be mounted at the fingers. Two straps attach the design to the hand: one at the fingers and one at the wrist. This adjustable strap can be stretched over the fingers in one movement with the anterior arm facing away, not requiring supination or extension of the fingers. The strap at the wrist attaches using an adjustable side release buckle.

### VTS Armband:

The VTS Armband (Figure 3b) was revised to be more narrow than its preliminary design, at 2 inches. At one end of the armband is a cinch buckle that allows the armband to be secured using one hand. The buckle frame is 0.75 inches wide. At the opposite end of the armband is a tapered point. Fabric is layered to make this tapered end rigid. This end secures using Velcro.

### VTS Palm:

The final version (V4) of the VTS Palm (Figure 4b) has a brace-like design that conforms to the palm using 1 inch foam embedded in between two fabric layers. This pad can be worn with the fingers extended and relaxed, or can be gripped by fingers that are flexed. Two straps attach the prototype to the hand. One strap at the wrist wraps around the arm and attaches using Velcro. A second strap stretches over the fingers.

### A. Themes

Four themes were taken directly from common codes, and two themes (#2 and #3) were formed in response to critical failures. All other codes simply stated if a prototype was good or bad regarding weight, fit, comfort, or donning. All codes and their occurrence per prototype are tabulated in the Supplementary Material.

#### 1) Hand Supination Is Challenging:

**Fig. 6.**
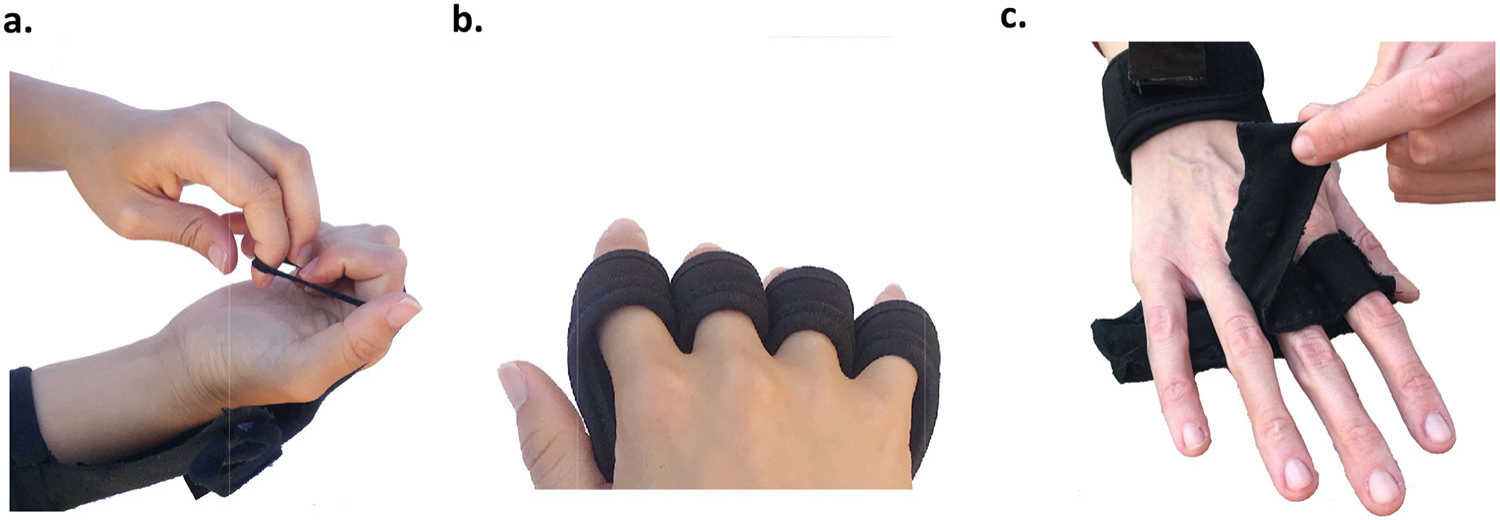
Design features that participants struggled with in this study. (a.) Participants struggled to supinate their arm and expose the volar hand to stretch loops over each finger. (b.) Participants could not insert their fingers into finger sleeves. (c.) When magnets were placed between the fingers, participants struggled with fit and finger abduction.

Five out of eight participants expressed that they had difficulty flipping over their hand to reveal their palm (supination). Proctors also noted that supination was impossible even for a participant with near normal function in their arm, P5. These comments were made in response to prototypes that include a strap that wraps around the limb, requiring the arm to rotate in pronation and supination (e.g. Figure 3a). These comments were also made in response to version V2 of the VTS Phalanx design which included loops that stretch over each finger (Figure 6a). This fixture encouraged donning in the supinated position particularly when the hand is curled (due to spastic hypertonia or contractures), making the fingers only accessible with the anterior arm exposed. For example, participant 4 stated:

“I can’t do the external rotation [supination].”

- P4

Instead of wrapping a strap around the arm, later prototypes included a cinch buckle to allow participants to tighten straps in a cinching motion using just one hand.

#### 2) Separate Finger Attachments Inhibit Fit and Use:

Two participants were unable to don a prototype in the study. In both cases, the prototype (VTS Phalanx V3 and VTS Palm V2) included individual attachments for each finger. All participants who were able to fully don the prototype expressed difficulty with the interaction. VTS Phalanx V3 used magnets between each finger that allowed the strap to “snap” on (Figure 6c). Large hands did not accommodate any fixtures between phalanges, and participants could not abduct their fingers to accommodate the magnets without help from the able hand. Participant 5 described this concisely:

“Spreading fingers is very difficult to do.”

- P5

VTS Palm V2 attached by resting the palm on the table and slipping each finger into a sleeve (Figure 6b). Though this design did not require the fingers to be held open (instead a flat surface extended the fingers), these sleeves required the fingers to be inserted. Participants, especially those with moderate weakness, struggled to slide their affected fingers or arm forward, which was necessary to insert the fingers to don this prototype. Participants who were able to fully don this prototype measured nearly double the donning time as the other versions of this device: Mean V2 = 71s (60-90s) vs. Mean V1 = 45 s (21-68s) and Mean V3 = 40s (16-66s) without experience). There was also a difference in donning time when donning with experience: Mean V2 = 64s (59-75s) vs. Mean V1 = 10s (9-11s) and Mean V3 = 30 s (25-35s).

#### 3) Fingers May Be Flexed or Open:

VTS Palm V1 (Figure 4a) was designed to be gripped within the palm since participants with spastic hypertonia may have fingers in tight flexion. Commercial braces use this form, such as the Hand Contracture Carrot Orthosis (AliMed, Inc.). However, spastic hypertonia varies in different physiological conditions [58] and participants explained how this variation in spastic hypertonia impacts device design. Participants mentioned that the fingers may either be open or flexed without the owner’s awareness, thus a rod-shaped device may not make reliable contact with the hand at all times. Subsequent prototypes of the VTS Palm (V2-V4) used a flexible, brace-like design that attaches to hands that are flexed or open.

#### 4) Fabric Coverage Impacts Comfort: Sweat:

In answer to questions about comfort, participants also discussed sweat. The VTS Phalanx design and the VTS Palm design were associated with some discussion on fabric coverage and sweat, including how it relates to sanitation.

“With the palm being one of the areas that is more sensitive to heat, sweat is a concern.”

- P1

#### 5) A Reduced Concern for Social Comfort:

5/8 (63%) of the participants made comments to the proctors suggesting that they have diminished concern for public opinion. This view was often restated – when asked about their level of social comfort while wearing the prototype, or about how they would design the device. Some participant comments include:

“I don’t care what people think when they see it. Maybe it’s me, I’m not a shy person.”

- P1

“Ever since this stroke, I stopped caring what people think when they look.”

- P5

“I’m not interested in what’s cool. Whatever works for my health, that’s cool.”

- P8

However, participants did make aesthetic requests. Namely, the request from four participants that the VTS Armband be more narrow so as to look more like a smartwatch. In addition, participants responded with lower ratings of social comfort when in public versus around friends and family. Wilcoxon signed-rank test shows that this difference is significant (Z=−2.52, p<0.05), and the skewness are different between the groups (Family: −1.7; Public: −0.85). No other nonsignificant tests were performed.

#### 6) The Affected Hand Is Infrequently Used:

All but one participant (7/8) in the study remarked that they do not use their affected hand. Such statements included:

“I can’t use this hand right now anyways.”

- P2

“I don’t do anything with this arm.”

- P8

## IV. Discussion

The VTS Glove successfully delivered wireless, wearable stimulation in an eight week clinical study. The hardware was modular, which allows the wearable device to be revised separately from the electronics.

The primary vulnerability of the VTS Glove was its form, which was difficult to don in the presence of impaired hand function. Measures like size and weight can be easily quantified, but accessible design requires further study and thus motivates the current work.

### A. Findings From the Current Study

In this study, we designed and revised VTS devices with accommodations for usability and wearability. The prototypes did not require extension of insertion of the fingers, and all designs were found to require less than two minutes of donning/doffing time. Most prior work articulates general needs and challenges for accessible wearable devices without investigating specific design features. In a review of needs for upper-limb assistive technology, design needs were summarized as “For stroke survivors and families, the devices needed to be easy to get on and off a weak and/or contracted hand/arm…and to be intuitive in terms of correctly positioning the device [59].” Durability, comfort, fit, weight, appearance, and cost were some other factors [50], [59], [60]. These other considerations mirror the principles of design for wearability, which we applied during the design of prototypes for this study [37], [52].

#### 1) Difficult Features and Potential Accommodation:

Supination of the forearm is a potentially common motion used in donning arm-mounted devices. Here, this motion was associated with straps that wrap around the arm, and attachments at the volar hand/anterior arm. Participants identified this motion as a potential challenge. At times, participants had to manipulate their affected hand using their unaffected hand. To expose the palm, participants had to grip and twist their affected arm with their unaffected hand; this left their able hand fully occupied. Straps with cinch buckles are one accommodation that prevents the need for this motion. Another potentially powerful design approach for this population is to require only that the affected arm be placed to rest on the device, and attachments can be made with no further movement from the affected limb. Participants can use their other hand to stretch open their affected fingers and rest the affected hand on the prototype (e.g. Figure 4b). Their unaffected hand is then free to secure the device on the dorsal side. Many braces adapt a cradle design that supports this procedure, but many other rehabilitation devices fail to.

Separate finger attachments were also found to challenge, and at times prevent, participants from donning prototypes. Results suggest that attachments which require insertion (e.g. VTS Palm V3, Figure 6b) or abduction of the fingers (e.g. VTS Phalanx V3, Figure 6c) are associated with increased interaction difficulty. As one participant noted in reference to VTS Palm V3, insertion of the fingers may also challenge individuals with affected range of motion at the shoulder, because the arm may need to slide the hand forward. Here we present one potential accommodation that provides accessible, separate attachments for each finger by using arches or indentations.

Results of our discussions about social comfort suggest that some individuals with stroke have a low level of concern for social comfort. However, the significant difference in ratings of comfort in public vs. with friends and family is notable. This difference suggests that social setting does influence comfort when wearing these prototypes. This influence could affect adherence and usage behaviors.

The final designs are all noticeable, but we do not expect this to dissuade individuals from using the device. In the clinical study, participants reported wearing the VTS Glove in social settings 19.1% of the time. In this study, ratings of social comfort were positive. To improve social comfort we can look to prior work: Shinohara *et al.* suggests that technology that supports the user’s ability (e.g. to do two-handed tasks) and desired identity (perhaps e.g. to have their fingers appear less contracted by spastic hypertonia, or with a device that appears like an accessory) is linked to better social accessibility of wearable and mobile assistive technology [61]. Future prototypes can also be smaller and more low-profile to reduce noticeability. Such changes may also reduce fabric coverage and increase physical comfort.

#### 2) Other Considerations:

All designs aim to leave the fingers free, and the VTS Glove, VTS Armband, and VTS Phalanx allow use of the affected hand while wearing the device. The VTS Palm may prevent some use of the affected hand, but aims to leave the fingers and thumb free. Future work can evaluate whether certain features encourage or dissuade use of the affected limb. Some individuals with stroke have very limited or no range of motion of the affected hand, and there would be no effect on hand use for these individuals. Stimulation may be well suited to this group, since it does not require movement. Our preliminary RCT found increased range of motion in the stimulation group [17]. Even small increases in range of motion could enable previously ineligible patients to progress to traditional exercises for the limb. Whether or not an individual finds the device to affect use of the hand, they may still choose to wear the device in ambient settings outside the home such as car rides, performances, and worship services. In the clinical study, some participants reported wearing the VTS Glove during activities outside the home (restaurants, walking in the park, fireworks celebrations) as well as tasks requiring use of the affected hand (riding a bicycle, working out at the gym). These activities exemplify some of the benefits of wearability.

It is known that contracted fingers and limbs can make dressing difficult due to their inability to stretch and flex; however, we noted that limpness of flaccid fingers also makes don and doff challenging. Most designs were associated with high percentages of correct donning, and low ratings of confusion on bipolar scales. However, novel features such as magnets were confusing to participants. Designs that included individual finger sleeves or attachments between the fingers were not able to fit all participants. Buckles helped participants tighten the device. Final designs are ambidextrous. Proctors observed little strain of the prototypes, perhaps because these designs did not require insertion of the fingers or limb. The VTS Glove from the first study required repeated tugging and stretching that causes wear. Participants with limited dexterity have gross movements and therefore can cause device strain. More accessible designs may reduce interaction with the device and thus reduce damage.

Superiority of one design over another will be determined by the intended application and efficacy. Each of these devices is designed to provide stimulation to a different target on the upper limb, and thus each may be used for different clinical applications. The VTS Armband is intended for use when the muscle belly or tendon is the target of VTS, which is of particular interest in spastic hypertonia. The VTS Palm is designed to provide stimulation to volar hand, which may be the target in somatosensory impairments. The VTS Palm is also uniquely suited to have actuators embedded in the strap over the dorsal phalanx if combined stimulation is desired.

The current study’s sample size is a limitation. Although a larger sample size would be preferred, this study successfully provides initial data on patient interaction with the new VTS devices. Prior research employing iterative design includes similar sample sizes per round [62]-[64].

## V. Conclusion

The VTS Glove is a wearable device to provide vibrotactile stimulation to the affected hand in chronic stroke. In contrast to existing apparatus, the VTS Glove can provide stimulation for extended durations in a mobile design – to enable further study that expands on promising prior work regarding therapeutic vibrotactile stimulation. Based on the result of an eight-week study, adherence to this method of stimulation appears feasible in people with stroke. Participants each wore the device for over 140 hours, both at home and in social settings. The hardware was robust, but the design of the glove needed revision. Extension and insertion of the fingers was necessary to don the VTS Glove, and although these motions may be common in wearable device and garment interaction, they are a barrier for users with upper limb disability.

The current study revealed that arm supination is another donning motion that is difficult for individuals with stroke. Devices can be designed to avoid interaction with the anterior arm in view. Separate finger attachments, such as sleeves or loops, were found to require insertion of the fingers – once again challenging participants. Ratings of social comfort for the wearable VTS devices evaluated here were positive. This study also provided the opportunity for revision of the new device designs (VTS Phalanx, VTS Armband, and VTS Palm) which all can be donned in an average time of 48 seconds (vs. 5.05 minutes to don the VTS Glove).

## Supplementary Material

Supplemental material
