# Supplementary material for: Design of a Wearable Vibrotactile Stimulation Device for Individuals With Upper-Limb Hemiparesis and Spasticity
Source: IEEE Trans Neural Syst Rehabil Eng. Author manuscript; Available in PMC 2023 Apr 28. (PMC10139869; doi:10.1109/TNSRE.2022.3174808)
Supplement: Supplemental material [file NIHMS1890406-supplement-Supplemental_material.pdf]

Supplementary Materials: "Design of a wearable vibrotactile stimulation device for individuals with upper-limb hemiparesis and spasticity."

Caitlyn E. Seim, Member, IEEE, Brandon Ritter, Thad E. Starner, Member, IEEE, Kara Flavin, Maarten G. Lansberg, Allison M. Okamura, Fellow, IEEE

May 10, 2022

1 VTS Glove: Engineering

Design

Flexfit Lifting gloves (Harbinger, Implus LLC.) were adapted to provide the base fabric for the prototype. The glove was fingerless for fit, and palmless for sanitation and to allow wearing while operating a manual wheelchair [1]. The device was also lightweight, and can be used wirelessly. The appearance resembled exercise gloves, a recognizable design that may reduce confusion regarding how to wear the device correctly.

Electronics

Small vibration motors attach inside each finger sleeve of the glove at the dorsal proximal phalanx. We selected coin-shaped, eccentric rotating mass (ERM) motors (Model #310-113, Precision Microdrives) to produce 210 Hz vibration (measured in a laboratory setting for validation at 1.3 g and 175 Hz when attached to the glove). Stimulation settings vary by individual study aims. This frequency was chosen for activation of the cutaneous mechanoreceptors, specifically the Pacinian corpuscles, which respond to both direct vibration and vibration transmitted through the body at a frequency range of at least 10-400 Hz (preferentially responding at about 250 Hz) [2]. The glove stimulates each finger by activating the attached motor. Reinforced wires run from these actuators to a circuitry box.

The circuit board contains a microcontroller, power regulator, USB port, and lithium polymer battery charging circuit, microSD card slot, motion-sensing chip, and motor-driving circuit. The microcontroller (Feather M0 Adalogger, Adafruit Industries) runs the device via preprogrammed software. The development board includes an onboard power regulator chip that ensures constant voltage of 3.3V. Thus, even when the battery runs low, stimuli for the patient remain uniform. A 50 mAh lithium polymer battery and charging circuit allow users to plug the glove into the wall to charge, then use the glove wirelessly for up to four hours. A triple axis gyroscope (L3GD20H, STMicro) could detect vibration and movement, and logged this data to the native SD card. Data captured on the device was limited to timestamps and movement data. Security was handled by anonymization. Networking (such as Wifi or Bluetooth) was not part of this design. The motor-driving circuit used Darlington transistor array chips (ULN2003, Texas Instruments Inc.) to draw current from the battery. This circuit is suitable for ERM-type motors. The electronics interface was a tactile switch that turns the stimulation on or off.

A 3D-printed box housed the glove's control circuitry and secured it to the back of the glove using Velcro (Figure 1a in the main text). This box rested on the rigid metacarpal bones of the hand, so as not to impede bending of the knuckles or wrist. The average length for the smallest adult metacarpal bone (pinky) is 2.12 inches [3]. The hardware components within the box have a footprint of 2.5 in. $\times$ 2.0 in.

2 VTS Glove: Initial Usability Findings

VTS Gloves were given to individuals with stroke to use at home for eight weeks. This experiment revealed essential data on device design and usability for future development of the VTS Glove and other VTS wearable devices.

Participants were sixteen individuals with chronic stroke (ages 28-68, 1-13 years post stroke, 11 male/5 female) with impaired tactile perception and range of motion their hand. Active range of motion for this group was: MCP flexion 0°-78° (Mean=28°), MCP extension from flexed 0°-43° (Mean=11°), wrist flexion 0°-62° (Mean=15°), wrist extension from flexed 0°-58° (Mean=13°), elbow flexion 0°-145° (Mean=70°), elbow extension from flexed 0°-141° (Mean=44°). Participants were each given a glove and asked to wear it switched on for three hours daily for eight weeks while awake. The daily wearing time was chosen to be greater than prior work (0.5-2 hours), while not requiring a level of daily commitment from participants that could potentially discourage enrollment. The hours did not need to be consecutive. Participants were told that if the device was used less than 18 hours per week for two weeks, the participant would be counted as a drop-out. Although adherence under real-world conditions cannot be forecasted, this structure provides data on the potential for adherence

to the device and stimulation routine. Half of the participants were randomly assigned using a lottery system to receive vibrotactile stimulation from the device, while the other half had vibration disabled. Instruction matched for both groups and device appearance was the same. Participants were asked only to use the device and were blinded to the different conditions.

Adherence

The glove automatically recorded participation time each week as a measure of adherence. Participation time was measured as the amount of time when the device was switched on. The total time when the device was switched on *and moving* was also recorded when the change in gyroscope values surpassed a predetermined threshold, between vibrations every 10 seconds. These data were logged to the microSD card and examined during weekly study visits. Proctors recorded if each total was or was not between 18-24 hours for the week. All participants recorded at least 18 hours of usage time each week, and none surpassed 24 total hours in a single week. No difference was found between groups. Total time when the device was on matched the total time when the device was on *and moving,* suggesting that the glove was not set aside while switched on.

Hardware

Areas of damage were logged by proctors as a measure of durability. The VTS Glove circuitry remained intact and successfully produced mobile stimulation for the duration of the study. Damage recorded during the study was limited to breaks in solder joints on the peripheral wires leading to the vibration motors. This likely occurred when wires were pulled during the don and doff process. Any repairs were made during a study visit or the participant was given a duplicate device. Participants re-charged the devices daily using a wall adapter similar to other consumer electronics. Participants did not report confusion about how to wear and charge the device. The glove secured the vibration motors against the skin as validated by proctor visual inspections, but fit depended on custom sizes.

Activities

Participants were given a worksheet each week to record the activities they performed while wearing the VTS Glove. The worksheet contained seven sections for free-response notes, each labeled with a weekday. The second half of this worksheet included daily sections to record observations and comments. Participants were asked to write notes about device design, challenges, breakage, or anecdotes. Participants were also asked to record their self-reported daily wearing time on the worksheet. Responses on the worksheet were transcribed and analyzed by two independent raters. Themes were defined using affinity diagramming. Activities during VTS Glove use were associated with five themes which are reported in Figure 1b in the main text. Together, the themes of sitting and consuming media accounted for 59.6% of the reported activities. Some participants described wearing the glove to activities such as movies, church, brunch, family gatherings, riding a bicycle, exercising, and picnics.

Challenges with don and doff

Participants struggled to get the glove on and off their affected hand. Eleven participants reported difficulty with the don and doff process on their weekly worksheets. The VTS Glove could be worn when fingers are tightly gripped – there were no reports of discomfort, sores or pressure ulcers. In addition, the glove could remain securely attached at the wrist if the fingers are open. However, the don and doff process was a challenge.

Remarks

The VTS Glove successfully delivered wireless, wearable stimulation over eight weeks. Hardware dimensions were minimized to allow components to fit on the back of the hand, and the low-profile hardware was able to provide over four hours of battery life, enable sensing, stimulation, and data collection. Participants with vibration enabled on their device each received over 140 hours of stimulation. Adherence under real-world conditions cannot be forecasted based on these data. However, these data provide supportive evidence that such a device is tolerable for numerous hours with or without vibration enabled. Participants mostly reported wearing the device while doing restful activities, although they also reported wearing the VTS Glove to meals, movies, church, and other gatherings. These data suggest that the glove was mobile and socially comfortable. Some participants experienced significant difficulty when donning and doffing the VTS Glove.

3 Revisions and Evaluations of New VTS Device Designs

This section provides more details about each revision of the VTS prototypes from the main manuscript. Figure S1 also provides demographics for each participant in the design study.

VTS Phalanx design

Versions of this design all attached at the distal forearm and the fingers. Electronics can be mounted at the wrist or on the back of the glove, while vibration motors should be embedded in contact with each dorsal proximal phalanx. Laser-cut and 3D printed components formed the basis for all versions of this design, which eliminated the need to use a commercial component (previously the FlexFit lifting glove) as the base layer.

ID	Most Affected Side	Dominant Hand	Years Post-stroke	Age	Active Range of Motion (Flexion, Extension)						Modified Ashworth (Flexor)	
					MCP		Wrist		Elbow		MCP	PIP
P1	L	R	3	54	20	20	18	5	80	80	0	0
P2	L	R	0.5	50	10	0	30	0	35	0	2	3
P3	R	R	2	84	55	40	25	20	80	70	1	2
P4	R	R	4	48	20	0	0	20	50	30	0	0
P5	R	R	0.5	75	40	25	40	35	85	70	2	2
P6	L	R	3	60	40	0	0	0	10	0	2	1
P7	L	R	1	56	5	0	35	0	10	20	1	1+
P8	L	R	2.5	89	5	10	0	0	100	75	0	0

Figure S1: Participant demographics, voluntary range of motion in angular degrees, and Modified Ashworth scores for all participants. Voluntary flexion range was measured, not to include involuntary flexion from hypertonia. Voluntary extension range was measured from a flexed position.

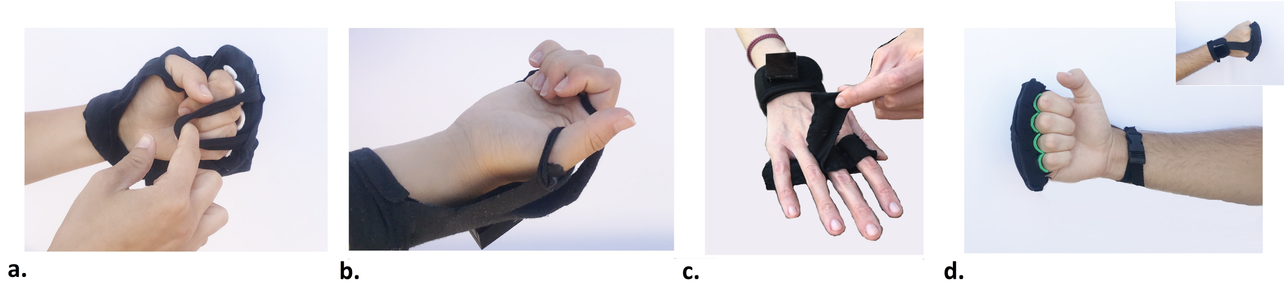

Figure S2: Versions of the VTS Phalanx device through rounds 1-3 and a final version. During the design study, the amount of fabric bulk was reduced and attachments were simplified to allow donning without forearm supination.

Round 1

This design shares the appearance of a fingerless, palmless glove. However, the fingers are attached using two flexible straps rather than individual sleeves (Figure S2a). These straps enable donning without extending the fingers. Each dorsal proximal phalanx fits into a rigid arch where actuators or sensors can be placed. Arches are mounted on a foam bar inside the garment that flexes to accommodate both large and small hands. The palm was kept uncovered for cleanliness.

Participants described this design as comfortable and secure, as shown in the table of thematic codes in Figure S5a. However, it was noted that the amount of fabric may lead to sweating.

"It felt like part of my hand." - P1

"This is probably the easiest glove I have tried to put on" - P2

Round 2

This version (Figure S2b) reduced the amount of fabric from Round 1 by making the fabric narrow and hand-shaped. This prototype attached to the hand using a loop over each finger. Rather than two straps (used in Round 1), five straps aim to make the design more intuitive. The stretchable loops were designed to be tight on the finger, while being able to entirely stretch over a contracted finger without requiring extension.

This version, even more so than the prior version, required participants to supinate their forearm while donning. Proctors observed that supination was impossible even for participants with near normal function in their arm. Two of the three participants in this round verbalized difficulty with this motion (Figure S5a).

"I cannot do the external rotation" - P4

Round 3

To eliminate the need for supination of the hand, the design was flipped to attach at the dorsal hand rather than volar hand. In theory, participants may stretch open their affected hand using their able hand, and place the affected hand to rest on the device. The weight of the affected hand then holds the device in place while the able hand secures the closures. To attach at the fingers, this design used magnets between each phalanx and allowed the strap to "snap" on. Figure S2c shows the magnetic strap midway through attachment or removal.

Though attachment using magnets required almost no movement from the participants, large hands simply did not accommodate any fixtures between phalanges. Participants considered the device comfortable, but also expressed that the design was "confusing" and "difficult to don" (Figure S5a). Using magnets as a fixture confused participants. On the Likert scale survey (Figure S6a), participants using this design agreed that "This device was confusing to don." Thus, in a final design, some features were restored from Version 2. Participants did not report difficulties related to supination when interacting with this design.

The magnet strap confuses me." - P4

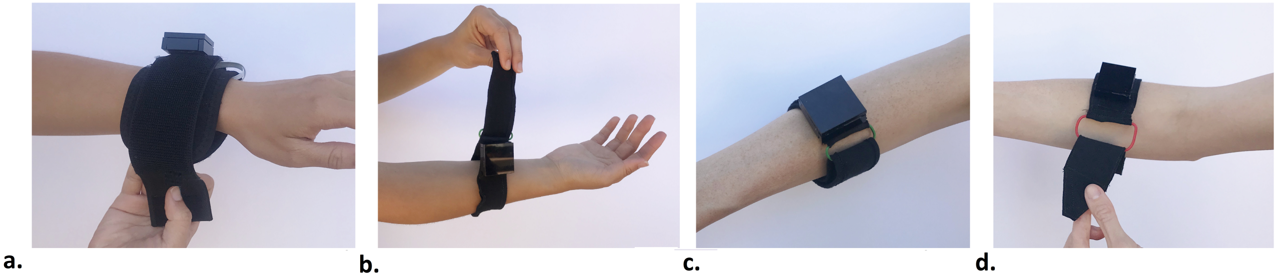

Figure S3: Versions of the VTS Armband through rounds 1-3 and a final version. A cinch buckle was added and enlarged to enable one-handed tightening of the device.

"Spreading fingers is very difficult to do." - *P5*

Final Design

In view of participant feedback, a final design was created. This version included the *least amount of fabric* to cover the hand – using a t-strap design to connect the fingers and distal forearm. To prevent confusion and *reduce steps in the donning process*, the design is attached to the fingers using a single strap. This adjustable strap can be stretched over the fingers in one movement, *not requiring supination or extension of the fingers.*

VTS Armband

This design was a band which wrapped around the forearm. Laser cut components form the basis of the prototypes. Electronics can be mounted along the band, where vibration motors should be embedded to apply stimulation to the forearm.

Round 1

This design consisted of a padded fabric band (3.0 in. wide) attached by a Velcro strap (total length: 15 in.). This length was chosen to accommodate arms of different sizes. Figure S5b displays participants' feedback about the armband design. According to interview feedback, participants struggled to supinate their arm when attaching the Velcro of this initial armband design. The orientation of the hook-and-loop was also a concern of participants when the abrasive hook-side of the Velcro touched the skin. Proctors observed that the armband would swing, fall or hit nearby furniture when the Velcro was released. Both participants mentioned an aesthetic request – that the strap be more narrow so the device could look more like a smartwatch.

"Make the band thinner. Then people might mistake it as a smartwatch." - *P1*

Round 2

This version was designed to be more narrow than Round 1 (1.5 inches). Like the prior version, the band attached using Velcro, and a cinch buckle was added. The objective of this buckle was to allow participants to tighten the device in a cinching motion using just one hand. This would eliminate the need to supinate the forearm. The buckle also made removal of the VTS Armband more easy: reducing the requisite strength of the Velcro needed to hold the device closed, and preventing the Armband from falling off the arm when the Velcro is released. Velcro no longer comes in contact with the skin using this design. When this design was tested, participants were not observed trying to supinate their forearm; however, participants reported difficulty when trying to insert the strap into the buckle.

"It was a little hard getting the end of the strap through the loop because it barely fits due to the width of the strap at the end." - *P3*

Round 3

This version was widened slightly (2 inches), which provides better grip on the arm to prevent the band from slipping down. The buckle's frame was made more accessible by expanding its size to 0.75 inches. Figure S3b-c shows the increased size of the buckle's frame. All participants reported comfort using this design, but one still reported difficulty inserting the strap into the buckle.

"Getting the strap through the buckle was like threading a needle." - *P6*

Final Design

A final design was made after the third round of participant feedback. This version (Figure S3d) maintains the 2-inch-wide band and large buckle design, while adding one accommodation. The end of the band was tapered and ridged; forming a point that could be more easily inserted into the buckle using one hand.

VTS Palm

This design is intended to be grasped or attached to the inside of the hand. The hand may be in a flexed position due to spasticity, thus, the VTS Palm device is either cushioned or flexible. When finalized, electronics can be mounted at the wrist, and vibration motors embedded to make contact with the palm.

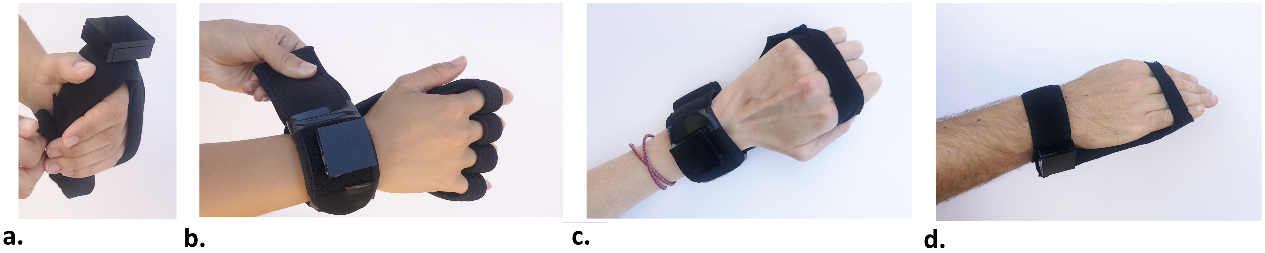

Figure S4: Versions of the VTS Palm device through rounds 1-3 and a final version. A graspable rod form factor was changed to a brace-like form factor that is compatible with clasped or relaxed hands.

Round 1

This version is designed to be gripped within the palm. The device has two main parts: the padded rod and the elastic strap. The padded rod consists of a plastic tube embedded in a foam bar, to provide a combination of rigidity and comfort. The elastic strap secures the device to the hand. Participants can place their affected hand over the foam bar and adjust the strap.

During the study, participants agreed that this design would get in the way of activities (Figure S5c). They also described that, due to changes in spasticity, their hand may be either open or flexed without their awareness throughout the day. Thus, this design may slip out of their grip.

"It kind of puts this hand out of commission." - P1

Participants also discussed sweat.

"With the palm being one of the areas that is more sensitive to heat, sweat is a concern." - P1

Round 2

To prevent the prototype from slipping off the hand, this version used a semi-flexible brace-like form factor that is compatible with both flaccid and flexed hands. This design did not include significant bracing structure, but one could reinforce the design to splint the hand. The donning process is very similar to the prior design: the participant opened the fingers of the affected hand with their able hand, placed their affected hand over the prototype and secured the attachments. The prototype was attached at the distal forearm with Velcro. The fingers slip into loops attached to the top of the prototype.

During the study, participants struggled to insert their fingers in the loops. These loops did not fit the fingers of some; participant P3 was unable to don this device and provide feedback on its comfort. Participants with flaccid paralysis struggled to slide their affected fingers or arm forward, which was necessary to don this prototype.

Round 3 and Final Design

This design shared the brace-like form factor of the prior version, but replaced the individual finger attachments with one flexible strap. This strap aimed to eliminate challenges with fit on different sized fingers, and allow participants to stretch over all the fingers without much manipulation. Feedback on this design was more positive, with all participants in this round reporting that it was easy to don with or without experience (Figure S5c). One participant discussed that the device could feel more securely attached, and this was reflected in their ratings of physical comfort on the Likert scale survey (Figure S6b). The final design made few changes from this version.

References

[1] L. T. Estes, D. Backus, and T. Starner, "A wearable vibration glove for improving hand sensation in persons with spinal cord injury using passive haptic rehabilitation," in *Proceedings of IEEE Pervasive Computing Technologies for Healthcare (PervasiveHealth)*, 2015, pp. 37–44.

[2] R. T. Verrillo, A. J. Fraioli, and R. L. Smith, "Sensation magnitude of vibrotactile stimuli," *Perception & Psychophysics*, vol. 6, no. 6, pp. 366–372, 1969.

[3] B. Alexander and K. Viktor, "Proportions of hand segments," *International Journal of Morphology*, vol. 28, no. 3, pp. 755–758, 2010.

	a. VTS Phalanx			b. VTS Armband			c. VTS Palm		
	1	2	3	1	2	3	1	2	3
Device is easy to don	50%	100%		100%	67%	67%	100%		100%
Device is secure	100%	33%		100%	67%	67%	50%	33%	33%
Device is lightweight		33%	33%	50%	67%	33%	50%	33%	
Device is comfortable	100%	33%	33%	100%	100%	100%	100%	67%	33%
Device would not limit activities	100%	67%	67%	100%	100%	67%		33%	33%
Comfortable in public	50%	67%	100%	50%	100%	100%	50%	33%	100%
Device is difficult to don	50%		67%		33%	33%		33%	
Device is confusing to don	50%		100%	50%	33%	33%	50%		
Device is too bulky		67%			33%			33%	
Device may cause sweating	50%	33%	33%				50%	33%	
Uncomfortable in public		33%						33%	
Difficult to supinate arm		67%		100%					33%
Design change: change strap colors for visual clarity							50%		33%
Design change: reduce band thickness				100%	33%	33%			
Not concerned with public opinion	50%	33%	100%	50%	33%	100%	50%	33%	100%
Affected hand is rarely used		33%	67%			33%	50%	67%	

Figure S5: Interview codes for each design across all three rounds. A colored block indicates the fraction of participants who made a response including that code. Codes found two or more times throughout the study are included. Codes are grouped into three colors for presentation clarity: positive codes (green), negative/challenge codes (red), and codes about lifestyle (blue).

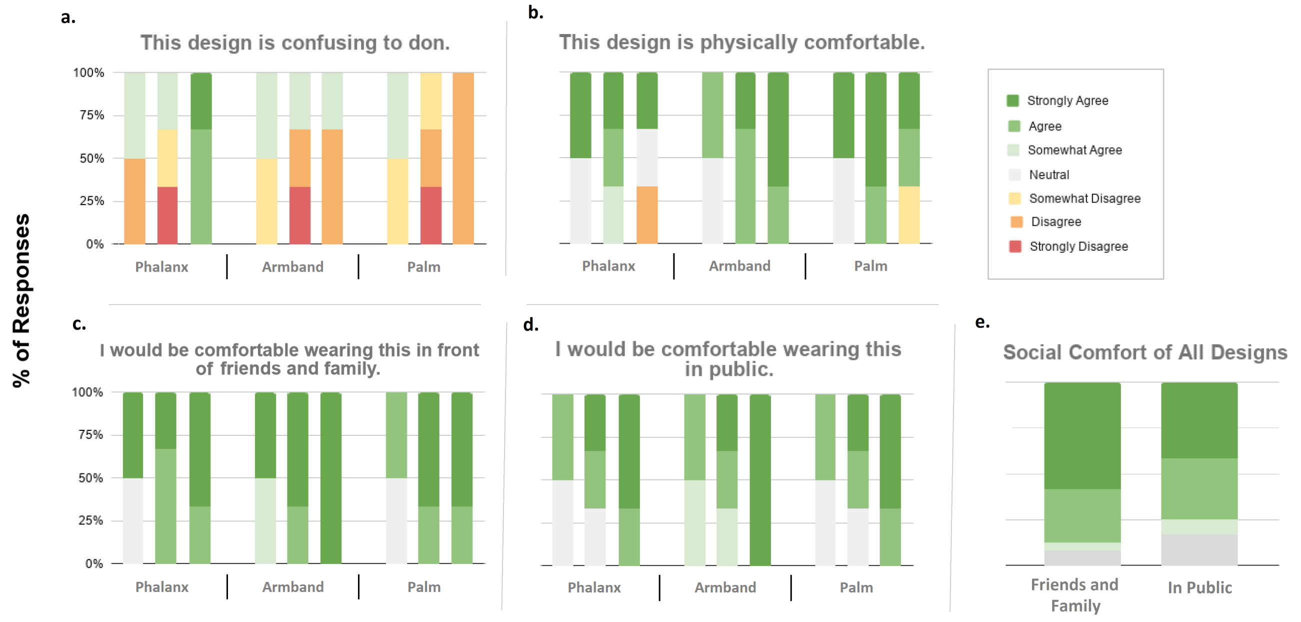

Figure S6: (a.-d.) Likert scale ratings for each device over three rounds. Each vertical bar represents one round. (e.) Total responses compared between statements about social comfort (“I would be comfortable wearing this [device] in front of...”).
